# Synthesis, In Vitro Testing, and Biodistribution of Surfactant-Free Radioactive Nanoparticles for Cancer Treatment

**DOI:** 10.3390/nano12020187

**Published:** 2022-01-06

**Authors:** Carla Daruich de Souza, Angelica Bueno Barbezan, Wilmmer Alexander Arcos Rosero, Sofia Nascimento dos Santos, Diego Vergaças de Sousa Carvalho, Carlos Alberto Zeituni, Emerson Soares Bernardes, Daniel Perez Vieira, Patrick Jack Spencer, Martha Simões Ribeiro, Maria Elisa Chuery Martins Rostelato

**Affiliations:** Nuclear and Energy Research Institute, IPEN-CNEN, São Paulo 05508-000, Brazil; angelbbarbezan@gmail.com (A.B.B.); arcosquim@gmail.com (W.A.A.R.); Snsantos@usp.br (S.N.d.S.); vergacas@gmail.com (D.V.d.S.C.); czeituni@ipen.br (C.A.Z.); ebernardes@ipen.br (E.S.B.); DPEREZV@usp.br (D.P.V.); pspencer@ipen.br (P.J.S.); marthasr@usp.br (M.S.R.); elisaros@ipen.br (M.E.C.M.R.)

**Keywords:** nanobrachytherapy, cancer treatment, radiation therapy, in vivo testing, in vitro testing, biodistribution, gold nanoparticles, gold-198

## Abstract

New forms of cancer treatment, which are effective, have simple manufacturing processes, and easily transportable, are of the utmost necessity. In this work, a methodology for the synthesis of radioactive Gold-198 nanoparticles without the use of surfactants was described. The nuclear activated Gold-198 foils were transformed into H^198^AuCl_4_ by dissolution using aqua regia, following a set of steps in a specially designed leak-tight setup. Gold-198 nanoparticles were synthesized using a citrate reduction stabilized with PEG. In addition, TEM results for the non-radioactive product presented an average size of 11.0 nm. The DLS and results for the radioactive ^198^AuNPs presented an average size of 8.7 nm. Moreover, the DLS results for the PEG-^198^AuNPs presented a 32.6 nm average size. Cell line tests showed no cytotoxic effect in any period and the concentrations were evaluated. Furthermore, in vivo testing showed a high biological uptake in the tumor and a cancer growth arrest.

## 1. Introduction

Cancer continues to be a devastating disease. The implications for the sick, the family, and society are, at best, pain and suffering. Even with the technological advance, there is a high probability of disability and death. According to the 2020 data collected by the International Agency for Research on Cancer (IARC), 20 million new cases and 9.9 million deaths were caused by cancer [1]. Possible treatments include surgery, radiation therapy, chemotherapy, hormone therapy, immunotherapy, and targeted chemical agent therapy [2]. New forms of treatment, which are effective, have simple manufacturing processes, and easily transportable, are of the utmost necessity. Of those forms, brachytherapy, a modality of radiation therapy that uses a radioactive source placed in or close to the target, is ideal, especially due to its portability and non-expensive equipment.

When developing a new radioactive therapy agent, a few practical issues should be considered. In addition to the isotope characteristics, such as energy, decay mode, and half-life, a study of the reaction feasibility within the radioactive constraints should be performed. For example, if the synthesis involves heating, extra steps should be made to simplify glassware (less radioactive waste), and radiation leakage of the setup have to be thoroughly investigated. Even chemical production routes, which might use reactants that do not necessarily change the outcome but add extra factors to the reaction, should be avoided [3].

Nanotechnology or particles that are manufactured in the nanoscale size, is a multidisciplinary scientific field that studies and creates products based on the tunable biochemical proprieties which occur in the nanometric size. In 2008, Katti et al. [4] started the discussion regarding radioactive nanoparticles (NPs) for cancer treatment. Unlike the classical radiation therapy, which is dependent on expensive machines, such as linear accelerators, ciber-knife, etc., the main advantage of nanobrachytherapy is low cost and portability, since the material is delivered by a syringe. This indicates that the treatment can go to the patient, and not the other way around.

In this scenario, this paper presents a surfactant-free synthesis route to produce radioactive Gold-198 nanoparticles. Then, the cytotoxicity of free NPs on different cell lines and developments was checked. Herein, the new treatment was tested in a murine model of prostrate tumor, in order to investigate the suitability of radioactive ^198^AuNPs for use in cancer treatment.

### 1.1. Radiation Therapy and Biology

Radiation interacts with materials that transfer its energy. This energy is attenuated by excitation or ionization. In the excitation process, radiation interacts with atoms, but not powerfully enough to remove electrons. Energy can be dissipated in the lattice in the form of phonons or it can create a knock-on-atom. In the ionization process, energy is considerably high to interact with the electron sphere and remove electrons. The electron-positive atom pair originates several secondary interactions [5].

In the human body, radiation mainly provokes water radiolysis, resulting in highly reactive species that can originate DNA and cellular damage. Moreover, radiation can directly collide with the DNA strand resulting in mutations [5]. The effects caused by radiation depend, basically, on tumor vascularity together with the type of radiation, concentration, and characteristics of radioactive material, as well as water and oxygen concentration [2].

Even with all of the technological advances, modern radiation therapy has a large amount of issues to consider. The radiation dose deposited in the target area (TA) must be directed to the TA, sparing the nearby healthy, non-affected tissues. Moreover, it should be sufficiently large to yield substantial cellular damage to cause cell destruction. Dose constraints are used to guide treatment plans. Created by specific associations, such as the American Association of Physicists in Medicine (AAPM), they are in continuous review. Undeniably, since brachytherapy places radioactive sources close to or inside the TA, it has a better control to yield fewer side effects [6,7].

If a radiation treatment could specifically penetrate and be retained inside tumor vascularity, it would increase cell damage without harming healthy tissues [2,8]. This is precisely what radioactive nanoparticles could achieve. The combination of brachytherapy with nanotechnology was expected.

Cancer cells require an extra blood supply and, ultimately, metastasize into other organs. To attain this, the cells promote the growth of vascular networks in a process called angiogenesis [9]. Without an adequate blood supply, cancers might turn necrotic [10,11]. An NP that is radioactive would effortlessly infiltrate tumor vascularity, carrying the radioactivity and its effects to the TA. Radioactive NPs tie both treatments: They use the harmful effects of radiation to kill cancer cells and cause the destruction of tumor vascularity. All of this happens from the inside-out, since they are small enough to freely roam inside the cancer’s blood vessels [12].

### 1.2. Radioactive Material

When developing a new radioactive treatment, characteristics such as energy, decay route and type, dose rate, and isotope concentration have to be suitable. They should not be a danger to hospital staff, production operators, and the public. In the case of operations needing hefty shielding, the final treatment price will be impacted by the higher manufacture costs [6,13].

Chemical stability, isotope and decay product form (solid, liquid or gaseous), and waste management have likewise a central role. The radioactive material needs to maintain its integrity inside the body or inside the outer encapsulation. For example, if a radiation source is made of an isotope that decays to a gaseous product, it is possible that the additional pressure might compromise the source integrity, resulting in a leakage [3,6,13,14].

### 1.3. Gold Nanoparticles (AuNPs)

To successfully synthetize AuNPs, the aggregation barrier increases resulting in an ideal NP size, allowing colloidal stability [15]. Indeed, radioactive NPs that are aimed at the cancer treatment are mostly synthetized in an one-step chemical reduction due to its simplicity, basic setups, and a non-toxic chemical reducing agent [16].

On the surface, since only one side of the NPs is bound to internal atoms, external sites are free to bound with donor-acceptor species. This results in the necessity of stabilizing NPs with suitable coordinating species, such as organic reducing agents (citrate and ascorbic molecules), thiols, surfactants or even biomarkers (serving both purposes) [17,18]. Moreover, the surface charge tuning from a negatively to positively charged surface is possible in order to increase the binding molecules types, possibilities or combinations [17,18].

Surfactants have a major impact on the NPs morphologies, since they can control the crystal growth of nanomaterials. Since morphology is also related to function, its application is impacted [19]. When the surfactants are added to a synthesis at a low concentration, they are amphiphilic substances that can adsorb into surfaces or interfaces, resulting in a modification of the available free energy [18,20]. The synthesis with surfactants usually uses a two-phase process in the presence of organic thiols. This route requires a more complicated setup, a large amount of control, a thorough investigation to ascertain reactant quantities, and, more worrisome, the handling of toxic materials. An increasingly simpler route is the stabilization by common reducing agents, such as sodium citrate and ascorbic acid. By mixing the original NP batch, which is stabilized weekly by the reducing agent organic portion with stronger binding agents, such as polyethylene glycol-PEG or arabic gum (GA), a replacement occurs that guarantees the integrity of the NP [18,21]. This is crucial for the stability tests in cells and, later, in the human body.

Gold atoms have three possible oxidation states: Au^+3^, Au^+1^, and Au^0^. The synthesis of AuNPs occurs with a reduction reaction, commonly with borohydride, amines, alcohols, carboxylic acids, sodium citrate, sodium borohydride, ascorbic acid, etc. [18,22,23]. The most common precursor used in the AuNPs synthesis is tetrachloroauric acid, HAuCl_4_ (in Au^+3^ state). This route will usually result in spherical NPs [18,24,25].

### 1.4. Examples of Radioactive ^198^AuNPs

Gold-198 in colloidal form has been used since the 1950s. P. Hahn from Meharry Medical College pioneered the use of Gold-198 to treat leukemia, for example, in [26,27,28].

For the fast manufacture of radioactive Gold-198 with higher yields, an isotope production nuclear reactor is required. Gold (almost 100%) is positioned in small canisters called rabbits. The rabbits are inserted into tubes leading to the reactor’s core, which are irradiated by the reactor neutron flux. The nucleolus of the atoms in the samples becomes instable (radioactive) when hit by the reactor neutron flux. Stable Gold (mass = 197) is activated into the radioactive Gold-198. Subsequently, the release of mainly beta radiation (≈314.6 keV) and Gamma (≈411.8 keV) have become a stable Mercury (mass = 198) [29].

The pathway to develop radioactive NPs must also allow for the procedure to be reproduced non-radioactively. Due to radioactive contamination, most of the necessary characterization equipment cannot be used. If the equipment is available, it must be in a controlled area with restricted access and must respect the radiation protection constraints.

It is clear that the research groups working to develop nanobrachytherapy are required to have a combination of backgrounds that are not easily found. From radiochemistry and radiation safety to materials science and radiobiology, the skill set crosses through many fields of physics, chemistry, biology, and engineering. Multidisciplinary teams and laboratories are necessary. For this reason, only a few groups are in this field.

Chanda et al. developed an ^198^AuNP coated with GA [23] for liver cancer treatment. The authors used high purity Gold foils activated to Gold-198 in a nuclear reactor. A trimeric alanine was specially developed as a reducing agent and GA as a stabilizer. Their final yield was ≈ 80 nm particles. In vitro tests confirmed the biological stability and hemocompatibility with no platelet aggregation. The treatment efficacy was tested in normal, SCID mice. NPs with 408 μCi (15.1 MBq) were injected into cancer induced mice by PC3 prostate cancer cells line. The treatment proceeded for 3 weeks. Consequently, the authors observed a cancer reduction of up to 82% [23].

Djoumessi et al. developed a combined NP for cancer treatment, PEG-^103^Pd:Pd@^198^AuNPs [30] containing Gold-198 with Palladium-103. The balance between both isotopes was ^103^Pd ≈ 1.2 mCi (44.4 MBq) to ^198^Au ≈ 0.4 mCi (14.8 MBq). In addition, 2 and 6 mg of Gold/kg of mice body weight were injected. The authors stated that only a little portion of radioactivity will be transferred to the surrounding blood vessels, although is likely that this amount will not result in toxic effects. The NPs were vastly retained in the tumors (>75%). Nevertheless, considerable amounts of NPs were also found in the liver (≈16%).

## 2. Materials and Methods

### 2.1. Reactants for Synthesis

All of the reactants were used as they were purchased, without any further purification. Glassware setups and magnetic stir bars were thoroughly cleaned with aqua regia (HCl and HNO_3_, Labsynth 87/471 and 65/486, respectively, São Paulo, São Paulo, Brazil) 3:1 *v*/*v* and thoroughly rinsed with ultrapure water (18.2 MΩ, Merck, São Paulo, São Paulo, Brazil) before use. Aqua regia is highly corrosive and poisonous. It can cause severe burns to all of the body tissue, and may be fatal if swallowed or inhaled. In addition, inhalation may cause lung and tooth damage. Therefore, all of the usual safety protection equipment must be used, such as lab coats, redundant gloves, and goggles. Moreover, it should be handled in a certified fume hood with proper ventilation.

Special attention was given to the built setups that prevent radiation leakage. Considerable experience was obtained with the non-radioactive product before the radioactive reactions were performed. The same nanopure water was used to prepare the aqueous solutions. HAuCl_4_ was only handled with plastic spatulas to avoid corrosion.

For both aqua regia and radioactive materials, trial runs were performed until the tasks could be performed with confidence.

### 2.2. Nuclear Activation

**Figure 1:**
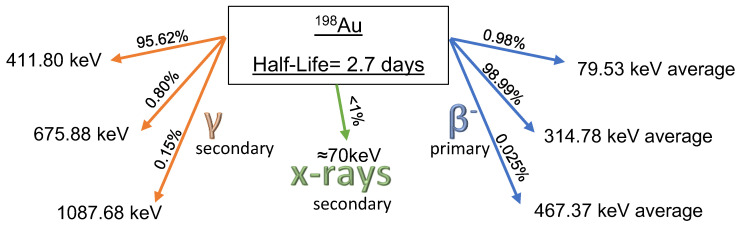
Gold-198 decay scheme.

Gold-198 is produced by neutron activation using the thermal neutron flux provided by a nuclear reactor. The pure material is placed inside aluminum capsules (known as rabbits) and travels to the core of the reactor by a pneumatic channel system. Natural Gold (197 mass) absorbs a neutron that turns into the radioactive Gold-198. It has a neutron cross section of 98.8 barns. Equation (1) shows the activation and decay. The decay scheme is shown in Figure 1.

(1) A79197u+n01 →activationA79198u+β−10+γ00→decayH80198g

Nuclear activation was performed in Gold foils (Goodfellow AU004925, disc thickness: 0.25 µm; specific density: 483 µg.cm^−2^; purity: 99.99+%, Huntingdon, England) at the IPEN IEA-R1 Research Reactor (Babcock & Wilcox Co., 5 MV, Charlotte, NC, USA). The final activity of an 8 h irradiation cycle was calculated using Equation (2) [31]. Confirmation of Gold-198 was obtained by an ORTEC HPGe detector.

(2) $$A=\frac{M\;\cdot \;N\;\cdot \;\theta \;\cdot \;\sigma \;\cdot \;\phi }{P}\;\;\left(1-e^{-\lambda t}\right)$$

where A is the activity (Bq), P is the atomic weight (g/mol), M is the mass (g), N is the Avogadro number, θ is the isotopic abundance in %, λ is the decay constant (time-1), ϕ is the reactor flow (n·cm^2^·s^−1^), σ is the cross section for the target atom (cm^2^), and t is time (s).

### 2.3. Synthesis of H^198^AuCl_4_

The following procedure was performed in the same way for the radioactive and non-radioactive precursor. Gold foils were transformed into HAuCl_4_ (or H^198^AuCl_4_) by dissolution using aqua regia (HCl and HNO_3—_Sigma) as follows:

- Gold metal disks with ≈0.7 g are placed in 50 mL of the special setup described below. Then, they are placed in 70 mL of aqua regia;
- The solution is progressively heated, and the final temperature is 50 °C. Within 1 min, the Gold will be dissolved in the solution, and the temperature will increase to 75 °C;
- Heat is maintained until the volume reaches ≈30 mL;
- Next, 10 mL of HCl is slowly added, which will remove the nitric component;
- The same temperature is maintained until it is concentrated to ≈30 mL;
- Steps 4 and 5 are repeated three times;
- The concentration is conducted by heating to achieve the final volume of 15 mL;
- The chloroauric acid precursor preparation is complete;
- A trap is used to control the pressure in the closed system.

A special compact setup was developed to perform the Gold-aqua regia dissolution, which has been proven to be a leak-tight radiation using the wipe test of all the junctions. The equipment was reused multiple times and it was stored in a lead box in between uses. It contained a pressure control glassware filled with liquid that is connected to a two-neck flask by bend connections. The ^197^Au disk is placed in the flask, which is transformed into H^198^AuCl_4_ by the addition of aqua regia in one of the flask openings. The other opening is where the pressure control device is connected. Furthermore, the flask is placed inside a heated equipment (“skirt” shape), and the heat is operated by an electronic controller device. 

### 2.4. Synthesis of ^198^AuNPs

Fifty milliliters of freshly prepared 0.1 mM HAuCl_4_ (or H^198^AuCl_4_) with 100 μL of 1 M NaOH (Casa Americana, 99.99%, São Paulo, São Paulo, Brazil) was prepared in a 100 mL three neck flask equipped with a reflux condenser. The solution was brought to boil (80–90 °C) under stirring. Thereafter, 5 mL of 34 mM sodium citrate was quickly added. The reaction turned from light yellow to clear, black, dark purple, to wine-red (2–3 min). The reaction proceeded for 7 min (10 min in total). The vial was removed from the heat plate and allowed to cool for 10 min under reflux. After this period, the reflux system was shut down and the DLS analysis was performed.

One milliliter of the NP solution was mixed with 0.1 mM of the polyethylene glycol (PEG-5000 MW, LaysanBIO, Arab, AL, USA) coating agent. The reaction was shaken in a microcentrifuge tube mixer for 2 h. The PEG and derivatives are highly biocompatible and are usually used as excipients in many materials for medical purposes [32]. It is a low-toxic, non-immunogenic, low molecular mass polymer that is soluble in aqueous solutions and organic solvents. Its properties make it a suitable choice for surface modification of NPs [33].

### 2.5. Characterization

Confirmation of Gold-198 production was obtained by an ORTEC HPGe detector (GEM-65970-B). The DLS (Anton Paar—Litesizer 500, Graz, Austria) analysis was performed with the radioactive product. TEM (JEOL JEM-2100F, Tokio, Japan) images were obtained with the non-radioactive product. The ImageJ software (National Institutes of Health, version 1.53k) was used to analyze the image and obtain size information.

### 2.6. Cell Culture

PC-3 (ATCC: CRL-1435) and LNCaP (ATCC: CRL-1740) prostate cancer cells were cultured in RPMI medium (Gibco, Life technologies, Baltimore, MD, USA), while the human umbilical vein endothelial cells (HUVEC) EA.hy926 (ATCC: CRL-2922) was cultured in DMEM medium (Life technologies, Baltimore, MD, USA). All of the cells were supplemented with a 10% fetal bovine serum (Life technologies, Baltimore, MD, USA) and antibiotic/antimycotic solution (Life technologies, Baltimore, MD, USA). The human prostate epithelial cell RWPE-1 (ATCC: CRL-11609) was cultured in Keratinocyte Serum Free medium (Life technologies, Baltimore, MD, USA) and supplemented with 5 ng.mL^−1^ of recombinant human epidermal growth factor (rEGF) and 0.05 mg/mL of bovine pituitary extract (BFE). All of the cells were maintained in a humidified incubator containing 5% CO_2_ at 37 °C.

### 2.7. Cell Viability Assay

A total of 2500 cells were seeded in a 96-well plate and incubated at 37 °C with 5% CO_2_ for 24 h. Next, the cells were incubated with and without AuNPs (volume ranging from 0.01 to 20 µL) for a period of 6 h. After this period, the medium was substituted and the cells were kept under culture for 48 or 72 h. Alternatively, the cells were incubated with and without AuNPs (volume ranging from 0.01 to 20 µL) for a period of 48 or 72 h The initial AuNP concentration was 2.43 × 10^11^ mL^−1^. Therefore, the concentration used ranged from 4.8 × 10^9^ to 2.4 × 10^6^ NPs corresponding to 20 to 0.01 µL, which is obtained by a serial 2-fold dilution. 

At the end of 48 or 72 h, the cells were fixed with 10% trichloroacetic acid at 4 °C for 1 h. Then, the plates were washed with tap water and air dried. Then, the sulforodamine B (SRB) solution (100 μL) at 0.4% (*w*/*v*) in 1% acetic acid was added to each well and incubated for 10 min at room temperature. Next, the plates were washed with 1% acetic acid and dried. Thereafter, 100 μL of 10 mM Tris base was added to the wells to solubilize the bound SRB, and absorbance was read at 515 nm on an automated microplate reader (Thermo Scientific, Nashville, TN, USA). Data were analyzed with GraphPad Prism 8.0 software. 

### 2.8. In Vivo Study

#### 2.8.1. Animals

The in vivo studies were conducted in male Balb/c nude mice (8 to 10 weeks). Balb/c nude mice were bred at the animal facility of the Nuclear and Energy Research Institute of São Paulo (IPEN). In addition, all of the experiments complied with the relevant laws and were approved by the local animal ethics committee (protocol number 243/19).

#### 2.8.2. Tumor Xenograft Models

For tumor induction, eight male Balb/c nude mice were implanted subcutaneously in the dorsal region with 2 × 10^6^ PC-3 cells diluted in 100 µL of PBS. Tumor growth was monitored for 25 days and measured with a digital caliper until the tumors reached a minimum size of 1.2 cm. Mice were observed twice a week for evidence of distress, ascites, paralysis or excessive weight loss.

#### 2.8.3. Treatment

When the tumors reached a size of 1.2 cm, Balb/c nude mice were divided in two groups (four mice per group). Mice were anesthetized with isoflurane (2%/O_2_) and then treated intratumorally with 30 µL of AuNPs (2.04 MBq), 7.2 × 10^9^ AuNPs or 30 µL of PBS (phosphate buffered saline). Tumors were measured with a digital caliper once a week for a period of 21 days. After the application, the animals remained under observation for a period of 21 days and tumor volumes were measured once a week with the aid of a digital caliper.

Three hours after the AuNPs intratumoral injection, one representative mouse was used for SPECT/CT imaging on an Albira µPET/SPECT/CT imaging system (Bruker Biospin Corporation, Woodbridge, CT, USA). The μSPECT/CT images were acquired under general anesthesia (isoflurane/O_2_) and heating at 37 °C. SPECT data were recorded via a static scan (FOV 80 mm, 60 s/projection) followed by a 20 min CT scan (FOV 80 mm, 35 kV, 400 µA). The μSPECT/CT scans were reconstructed with the Albira software (Bruker Biospin Corporation, Woodbridge, CT, USA) with ordered subsets expectation maximization (OSEM) and filtered back projection (FBP) algorithms, for SPECT and CT, respectively. In addition, the images were processed with the PMOD software (PMOD Technologies, Zurick, China).

At the end of the experiment, mice were euthanized and a pilot biodistribution study was performed for one animal. Organs of interest were dissected out and weighed for a quantitative estimation of Gamma counts using a Gamma counter (2470 Automatic Gamma Counter—PerkinElmer, Waltham, MA, USA), in order to quantify the percentage of injected dose per gram of tissue (% ID/g). The GraphPad 9.0 software was used to analyze the data and results.

## 3. Results and Discussion

### 3.1. Synthesis and Characterization

None of the aqua regia’s constituent acids can attack Gold alone. Nitric acid is a potent oxidant that dissolves an undetectable amount of Gold, creates the first cations, and initializes the reaction. Next, the hydrochloric acid’s chloride ions will strongly react, removing Gold from the dissolution. This allows the excess Gold to continue the process of oxidization. The high concentration of chlorides increases the solubility of metals, forming chloro-metallic coordination compounds, such as chloroauric acid. The appropriate equations (Equation (3)) are as follows:

(3) $$\mathrm{Au}+3{\mathrm{HNO}}_{3}+4\mathrm{HCl}\;↔{\;\mathrm{AuCl}}_{4}^{-}+3{\mathrm{NO}}_{2}+H_{3}O^{+}+2H_{2}O\;\mathrm{Au}+{\;\mathrm{HNO}}_{3}+4\mathrm{HCl}\;↔{\;\mathrm{AuCl}}_{4}^{-}+\;\mathrm{NO}+H_{3}O^{+}+H_{2}O$$

At the end of the process, the residual nitric acid was removed by repeated heating with the addition of hydrochloric acid. It can be visually accessed, since the nitric vapors use the litmus paper and have a brown color.

The wipe test was performed in the joints of both the precursor and the NP synthesis apparatus. They were measured by a Capintec CRC-15R well chamber and yielded no radiation count.

The learning and experience needed to execute this part of the project demanded time. Training and meetings with the nuclear reactor as well as the radiation protection staff were required. The transfer of the material from the reactor site to the destination laboratory was rehearsed for safety and efficiency. The setup, solutions, and equipment were ready 3 days prior to irradiation. Trial runs were conducted with cold (non-radioactive) materials 5 days prior to irradiation, in order for the steps to be fresh in the mind and memory of the operator. Finally, the process was optimized and fully completed in 3 days.

**Figure 2:**
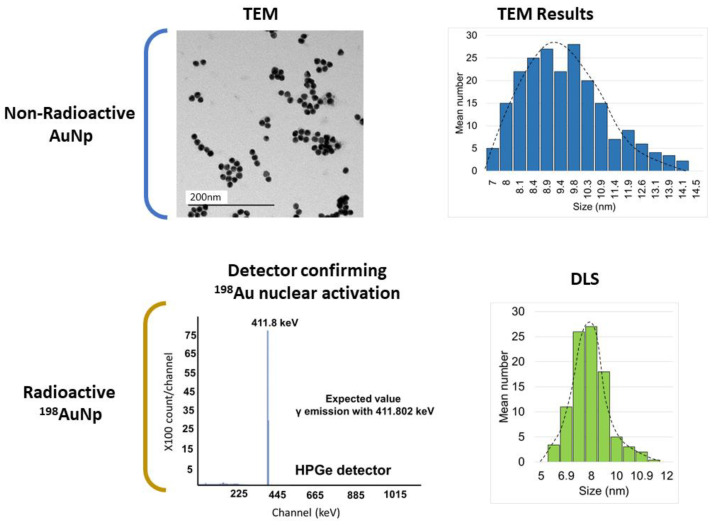
TEM results for the non-radioactive product (average size: 11.0 nm; median: 9.4 nm; standard deviation: 2.3 nm). DLS and HPGe detector results for the radioactive AuNPs (average size: 8.7 nm; median: 9.2 nm; standard deviation: 2.0 nm).

Figure 2 shows the characterization results. TEM results for the non-radioactive product showed an average size of 11 nm. The DLS measurement of the radioactive product presented a size of 8.7 nm. This small difference was expected. According to Souza et al. [34], the presence of dispersants can induce errors in the DLS measurements. The authors found up to 20% difference in comparison, which highlights the importance of analyzing the NP size by multiple techniques. The HPGe detector confirmed the presence of Au-198 after irradiation with no contaminants.

**Figure 3:**
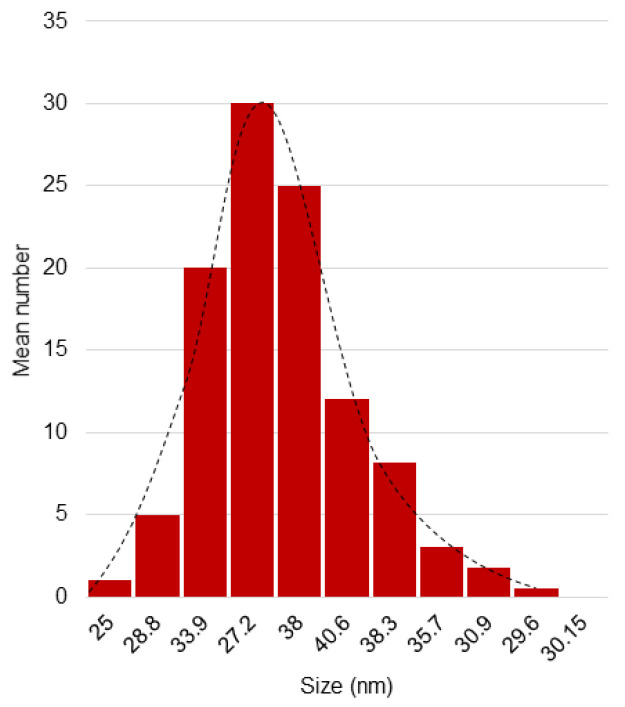
DLS results for the radioactive PEG-^198^AuNPs (average size: 32.6 nm; median: 30.9 nm; standard deviation: 5.1 nm).

Figure 3 shows the DLS results for the PEG-^198^AuNPs. There is an increase in size due to the complexation of the stabilizing polymer on the surface, but the final size is still in the nano range.

### 3.2. In Vitro Studies—Cell Viability

**Figure 4:**
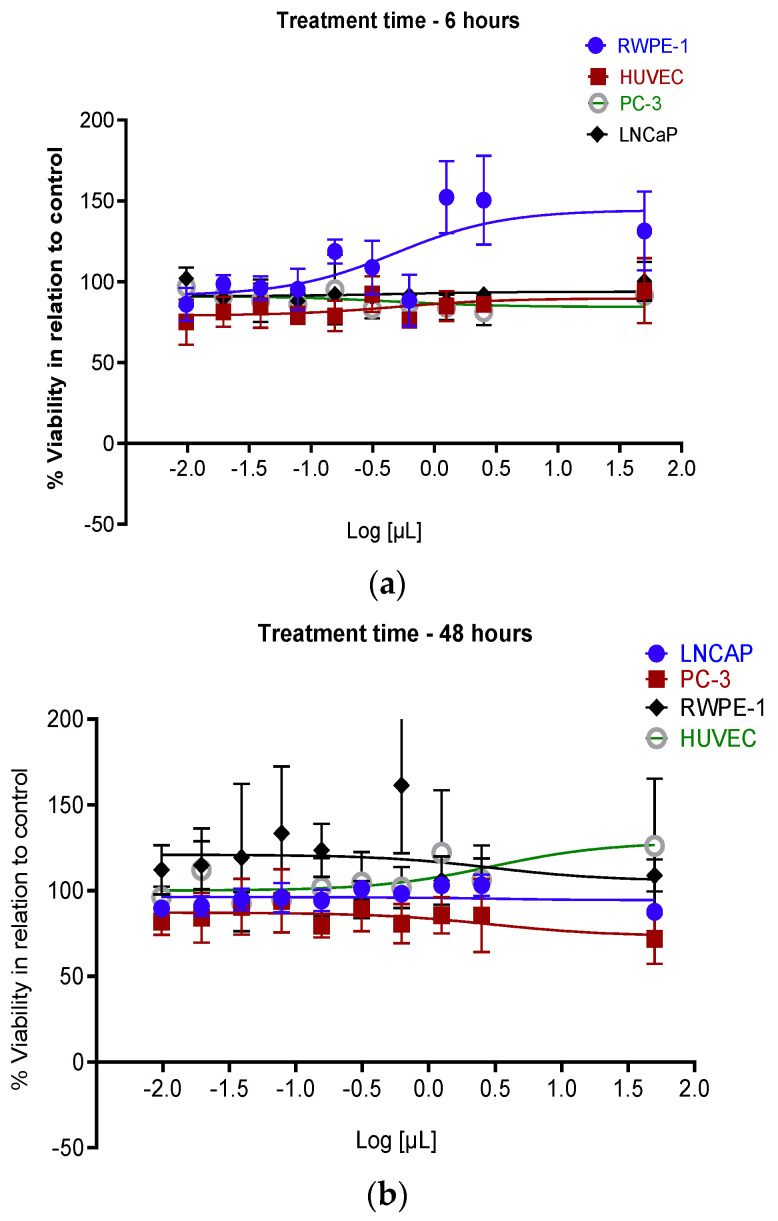

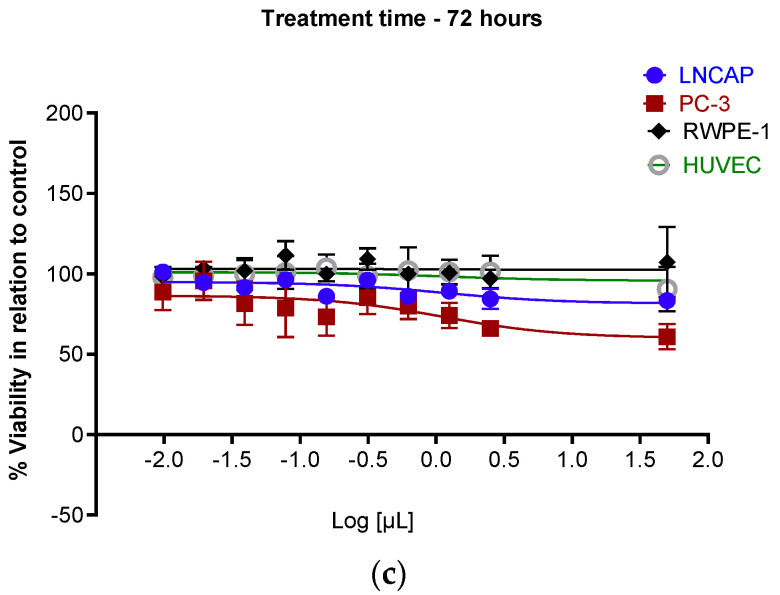
Sulforodamine B viability assay showing LNCaP, PC-3, RWPE-1, and HUVEC cells viability under the treatment of AuNPs for 6 h (**a**), 48 h (**b**), and 72 h (**c**), then cultured for an additional 72 h, were determined as a percentage of viable cells relative to control (cells with no NPs treatment). The volume used ranged from 0.01 to 20 µL corresponding to 2.4 × 10^6^ to 4.8 × 10^9^ AuNPs.

Figure 4 shows the effect of AuNPs on LNCaP and PC-3 prostate tumor cells, on the non-tumor line RWPE-1, and on the endothelial cell line HUVEC in periods of 6 (Figure 4a), 48 (Figure 4b), and 72 h (Figure 4c).

The treatment of LNCaP and PC-3 prostate cancer cells or the non-cancerous human prostate epithelial cell RWPE-1 and the human umbilical vein endothelial cells HUVEC with increasing doses of AuNPs (from 0.01 to 20 µL) showed that AuNPs had no effect on the cellular viability (Figure 4a–c). These data indicate that AuNPs are not toxic to cancer or healthy cells in vitro.

### 3.3. In Vivo Tests—Therapeutic Efficacy

**Figure 5:**
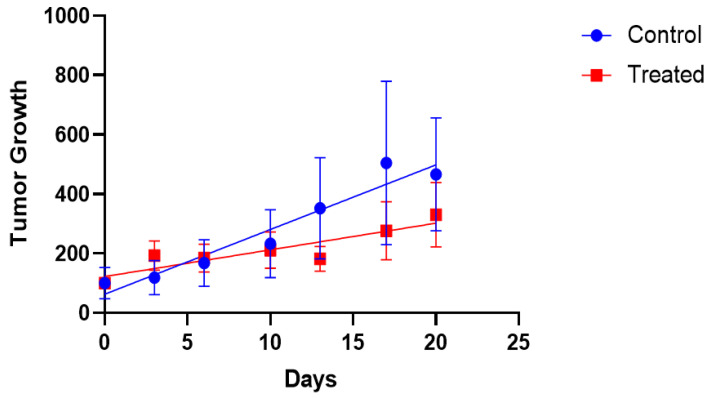
Evaluation of the effectiveness of in vivo treatment with radioactive AuNPs. Tumor size measured in mm^3^. Data are presented as mean values ± SEM (*n* = 4/group).

Figure 5 exhibits the tumor growth for both the treated and control groups. Despite some relatively large standard error of mean (SEM) values, the F-test showed that slopes from the control (21.77 ± 2.56) and treated groups (8.98 ± 2.021) are significantly different (*p* = 0.0029; F = 15.38, DFn = 1, DFd = 10). These data demonstrate that radioactive AuNPs are able to significantly arrest the tumor development over time.

### 3.4. In Vivo Tests—µPET/SPECT/CT and Biodistribution Studies

**Figure 6:**
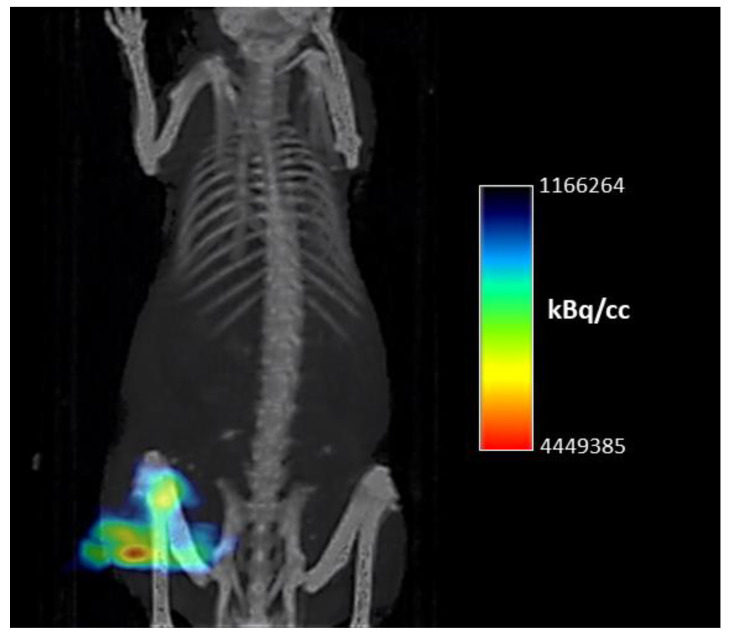
Representative static small animal SPECT/CT image 3-h after the intratumoral injection of 2.04 MBq of ^198^AuNP in the PC-3 tumor bearing Balb/c nude mouse. The image is displayed as the maximum intensity projection.

Static SPECT/CT image 3 h post-injection showed that ^198^AuNP primarily accumulated in the tumor site of Balb/c nude mice bearing PC-3 tumors (Figure 6). The ex vivo biodistribution analysis at the end of the treatment study confirmed that the SPECT/CT observation shows that the ^198^AuNP uptake was preferentially found in the PC-3 tumor. Moreover, we could observe some uptake in the liver and spleen of the mouse (Table 1).

## 4. Conclusions

In this work, we successfully developed a methodology for the synthesis of radioactive ^198^AuNPs without the use of surfactants. The methodology is simple and the radiation is leak proof. The NPs were characterized and tested by in vitro and in vivo studies. Nonradioactive NPs showed no cytotoxicity on tumor and normal cells. In vivo, radioactive NPs were able to arrest tumor growth. This initial result is very promising. We are currently evaluating this alternative treatment in a more thorough study that will uncover the correct treatment protocol, showing the relation between the concentration of radioactive NPs to cancer size, likewise accessing possible side effects with histological tests. In addition, we will test it in other animal models. Moreover, we are working on a dosimetric method through experimental and simulation (Monte-Carlo methods) routes. We hope this work encourages new research to advance the use of nanobrachytherapy in cancer treatment.

## Figures and Tables

**Table 1:** Biodistribution of ^198^AuNP 21 days after intratumoral injection in a Balb/c nude mouse bearing the PC-3 tumor.

Organ	% ID g^−1^	Organ	% ID g^−1^
Blood	0.04	Intestine	0.00
Heart	0.02	Pancreas	0.01
Lungs	0.04	Bone	0.04
Liver	0.63	Muscle	0.00
Kidneys	0.03	Brain	0.00
Spleen	0.13	Bladder	0.00
Stomach	0.00	Tumor	10.07
